# Role of clinical pharmacists in epilepsy management at a general hospital in Vietnam: a before-and-after study

**DOI:** 10.1186/s40545-021-00394-9

**Published:** 2021-12-20

**Authors:** Hong Tham Pham, Minh-Hoang Tran, Ngoc Quy Nguyen, Van Tan Vo, Manh Hung Tran

**Affiliations:** 1 Department of Pharmacy, Nhan Dan Gia Dinh Hospital, Ho Chi Minh City, Vietnam; 2 Department of Pharmacology, Faculty of Pharmacy, University of Medicine and Pharmacy at Ho Chi Minh City, 41–43 Dinh Tien Hoang Street, Ben Nghe Ward, District 1, Ho Chi Minh City, Vietnam; 3 Institute of Environmental Sciences, Nguyen Tat Thanh University, Ho Chi Minh City, Vietnam; 4 Department of Neurology, Nhan Dan Gia Dinh Hospital, Ho Chi Minh City, Vietnam

**Keywords:** Adverse drug reaction, Antiepileptic drug, Clinical pharmacist, Epilepsy, Vietnam

## Abstract

**Background:**

Clinical pharmacists have an important role in inter-professional healthcare collaboration for epilepsy management. However, the pharmacy practices of managing epilepsy are still limited in Vietnam, deterring pharmacists from routine adjustments of antiepileptic drugs, which could decrease the patients’ quality of life. This study aimed to assess the effectiveness of pharmacist interventions in epilepsy treatment at a Vietnamese general hospital.

**Methods:**

A before-and-after study was conducted from January 2016 to December 2018. All patients with a diagnosis of epilepsy and being treated at the investigated hospital were recruited and screened for eligibility and exclusion criteria. The primary outcome was the proportion of patients in good control of their epilepsy (with two seizures or less in a year). The secondary outcome was the number of patients maintaining optimized concentrations within the therapeutic range of carbamazepine (4–12 mg/L), phenytoin (10–20 mg/L), or valproic acid (50–100 mg/L). Collected data were analyzed using two proportions *Z*-test or Chi-square test.

**Results:**

A total of 141 participants were enrolled in the study. While most patients were given lower prescribed daily doses than the recommendations from the World Health Organization, over 56% of the participants still experienced adverse drug effects. More than half of the patients received at least one pharmacists’ intervention, which increased by 25.0% the effectiveness of the therapy (*p* < 0.001) and by 14.6% the number of patients with optimized drug concentrations (*p* = 0.018).

**Conclusion:**

Epilepsy management requires a multiple-stepped and comprehensive approach, with a focus on the health and safety of the patients. As part of the healthcare team, pharmacists need to engage at every stage to monitor the patient’s response and determine the most effective treatment with the fewest adverse drug reactions.

*Trial registration* ClinicalTrials.gov, NCT04967326. Registered July 19, 2021—Retrospectively registered, https://clinicaltrials.gov/ct2/show/NCT04967326

## Background

Worldwide, the prevalence of epilepsy is about 6.38 per 1000 persons, and annually, the incident rate is estimated at 61.44 per 100,000 person-years [1]. This figure could be higher in countries with low or average incomes (138.99 per 100,000 person-years) [1]. The current primary treatment involves antiepileptic drugs (AEDs), which aim to prevent seizures with the fewest adverse events. However, this general treatment approach tends to show unpredictable effectiveness, adverse drug reactions (ADRs), and sometimes, inappropriate dosages. Moreover, in clinic-based cohorts, over one-third of patients experience drug-resistant epilepsy [2].

Phenytoin, carbamazepine, and valproic acid, which are among the first-generation AEDs, are prescribed in many countries around the world [3], including Vietnam. These agents have complicated pharmacokinetics [4], which may result in alterations in absorption, distribution, and metabolism. This means that, given the same dose, the serum concentration of each drug may vary between patients. The management of epilepsy, as a result, requires inter-professional collaboration to ensure therapeutic optimization. As healthcare professionals, clinical pharmacists play an important role in epilepsy management, which includes establishing a therapeutic drug monitoring (TDM) protocol, adjusting doses, monitoring ADRs, etc.

However, the clinical pharmacy practices in epilepsy management are quite limited in Vietnam. The treatment gap—the proportion of people with epilepsy who are not adequately treated—still remains very high, especially in rural areas (84.7%), which probably results from discontinuing the treatment or refusing to take medications [5]. This shows a need for pharmacist consultations for patients with epilepsy and their family members, as they may be lack information about AEDs or motivation in controlling potential seizures. In addition, the adjustments of antiepileptic drugs by pharmacists are not routine procedures, nor are monitored for effectiveness in many Vietnamese hospitals. This lack of engagement threatens the patients’ safety and decreases their quality of life. To address this issue, certain interventions are needed to enable pharmacists to manage patients with epilepsy more systematically. This study was therefore conducted to evaluate the effectiveness of pharmacist interventions in epilepsy treatment at a general hospital in Vietnam.

## Methods

### Study design and participants

Nhan Dan Gia Dinh (NDGD) Hospital is a general hospital under the management of the Department of Health, Ho Chi Minh City, Vietnam, with about 1500 inpatient beds and 5000 outpatient visits per day. A before-and-after study was conducted in the Department of Neurology at NDGD Hospital from January 1, 2016 to December 31, 2018.

All patients with a diagnosis of epilepsy and being treated at NDGD Hospital from January 1, 2016 to January 1, 2017 were recruited. Patients were eligible if they (1) were prescribed a monotherapy or polytherapy of phenytoin (PHT), carbamazepine (CBZ), or valproic acid (VPA), and (2) were treated for more than one month. Patients were excluded if they (1) were pregnant or breastfeeding women; (2) had a history of alcoholism; (3) had liver or renal disease; and (4) were using drugs known to have an influence on cytochrome P450 (CYP450) enzymes.

The required sample size was calculated using the online tool Calculator.net [6], with a confidence level of 95%, 5% margin of error, and an approximate population size of 250 epileptic patients being treated at NDGD Hospital per year with either PHT, CBZ, or VPA. Following our survey on patients with epilepsy in 2016, without any healthcare interventions, about 63% had good seizure control. We estimated that for the pharmacist interventions to be clinically significant, at least 75% of the patients would need to have good seizure control by these implementations, resulting in a minimum sample size of 135 patients.

In the first period, before implementing the pharmacist interventions, patients were screened for demographic characteristics, drug serum concentration, and information about previous treatments. After the interventions, patients were followed up for one year, and at the study endpoint, patients were screened again for the study outcomes.

### Intervention

Following blood sample collection, the clinical pharmacist interventions were carried out, involving TDM for each patient to optimize their AED therapy. This optimization involved either medication consultation, dosage adjustment, medication switching/discontinuation, or combination therapy, based on the serum concentration of AEDs and the patient’s ADRs. Only patients whose blood samples or clinical manifestations revealed unoptimized regimens were assigned to the subsequent optimization interventions. All the physicians and pharmacists involved had reached a consensus on the relevant interventions.

### Outcomes

The primary outcome was the effectiveness of the AED therapy, measured by the number of seizures during the first year of the study and during the year prior to the study endpoint. Patients with two seizures or less in a year were categorized as having good control, whereas those who had more were considered to have poor control. The secondary outcome was the number of patients who maintained an optimized concentration of AED. The targeted therapeutic ranges for CBZ, PHT, and VPA were 4–12 mg/L, 10–20 mg/L, and 50–100 mg/L, respectively.

### Statistical analysis

Data collected from medical records were presented as means with standard deviation (SD) or median with interquartile range (Q1–Q3) for continuous variables or as frequencies with percentages for categorical variables. Differences in proportion were analyzed using the two proportion *Z*-test or the Chi-square test. Best–worst case analyses were also conducted to control for potential bias due to loss to follow-up. All hypotheses were carried out with a confidence level of 95%. The analyses were performed using the Statistical Package for Social Sciences software, version 25.0 (SPSS Inc., Chicago, Illinois, United States).

### Ethics approval

This study was approved by the NDGD Hospital Ethics Committee, Ho Chi Minh City, Vietnam, with approval number 23-2015/CN-HĐĐĐ, on October 13, 2015. The patients included all gave their informed consent.

## Results

### Demographic characteristics of the participants

**Fig. 1:**
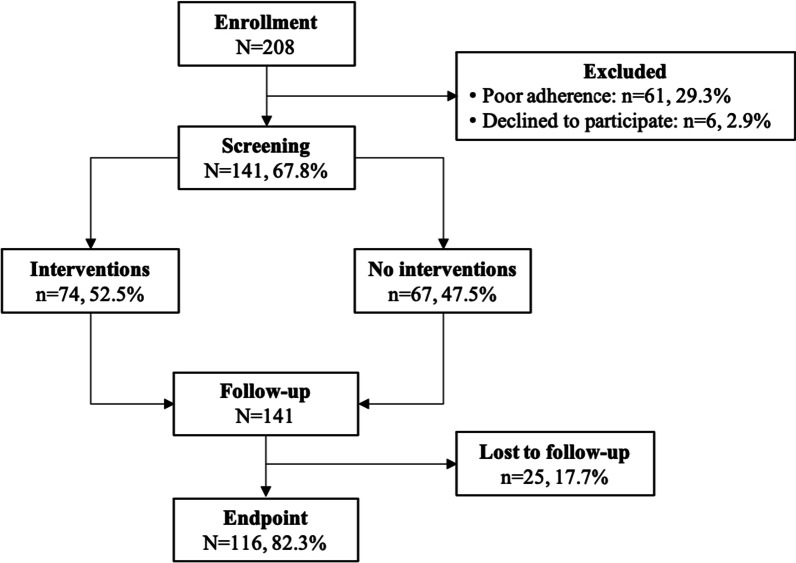
Flow diagram of the study participants

A total of 141 epileptic patients were enrolled in the study, as shown in Fig. 1.

The characteristics of the study participants are shown in Table 1. Of the 141 participants, the median age was 54, ranging from 16 to 81 years old. The median body weight was 58 kg, with 34.8% being overweight or obese. About 40% of the patients have undergone treatment with AEDs for more than 10 years. The most favored regimen was monotherapy, accounting for over 70% of the patients.

**Table 1:** Demographic characteristics of patients with epilepsy at the start of the study (*N* = 141)

Characteristics	Value (*N* = 141)
Gender, *n* (%)	
Female	59 (41.8)
Male	82 (58.2)
Age (years), median (Q1–Q3)	54 (38–61)
Body weight (kg), median (Q1–Q3)	58 (52–65)
Body mass index (kg/m^2^), mean ± SD	22.2 ± 2.8
< 23, *n* (%)	92 (65.2)
≥ 23, *n* (%)	49 (34.8)
Duration of treatment, *n* (%)	
< 5 years	68 (42.2)
5–10 years	24 (17.0)
> 10 years	49 (40.8)
AED therapy, *n* (%)	
Monotherapy	99 (70.2)
Polytherapy	42 (29.8)

*Q1–Q3* interquartile range, *SD* standard deviation, *AED* antiepileptic drug

### Characteristics of AED therapy

The defined daily dose (DDD) and prescribed daily dose (PDD) for AEDs are summarized in Table 2. It is evident that the average PDD for all three AEDs was much lower than the DDD recommended by the World Health Organization (WHO) [7], Phenytoin was the drug with the highest proportion of PDD/DDD in both monotherapy and polytherapy. Patients on monotherapy received a less dispersed and lower PDD compared to those taking multiple AEDs.

**Table 2:** Dosage of carbamazepine, valproic acid, and phenytoin at the start of the study

	Carbamazepine (*n* = 64)	Valproic acid (*n* = 65)	Phenytoin (*n* = 29)
DDD^a^ (mg)	1000	1500	300
Monotherapy, *n*	40	38	21
PDD^b^ (mg), mean ± SD	446 ± 216	669 ± 277	213 ± 46
%DDD^c^	44.6	44.6	70.8
Polytherapy, *n*	24	27	8
PDD (mg), mean ± SD	587 ± 456	972 ± 528	225 ± 50
%DDD	58.7	64.8	75.0

^a^DDD: defined daily dose, retrieved from https://www.whocc.no/atc_ddd_index

^b^PDD: prescribed daily dose

^c^%DDD = PDD/DDD

Table 3 describes observed ADRs that patients experienced during the study. More than 40% of the participants did not experience any symptoms indicating ADRs. In the remaining patients (56.7%), dizziness, headache, or sleep disorders were the most common adverse effects. Other ADRs were also reported, such as constipation, nausea, vomiting, confusion, hepatotoxicity, rash, or severe cutaneous adverse reactions. Specifically, the study recorded eight patients with HLA-B*1502, a known risk factor for CBZ-induced Steven–Johnson syndrome in Asian patients [8], out of 13 cases of cutaneous ADRs, leading to medication discontinuation in these patients. There was no statistically significant difference in the total number of patients with ADRs between the mono- and polytherapy.

**Table 3:** Adverse drug reactions to AED therapy (*N* = 141)

Adverse drug reactions (ADRs)	Monotherapy (*n* = 99)	Polytherapy (*n* = 42)	*p*-value
No symptoms of ADRs, *n* (%)	45 (45.5)	16 (38.1)	0.420^*^
Experienced ADRs, *n* (%)	54 (54.5)	26 (61.9)	
Dizziness or headache	20	12	
Sleep disorders	24	9	
Weight gain	7	3	
Depression	2	2	
Itchiness or rash	9	4	
Others	32	16	

*Chi-square test; the ADRs were recorded until there was a loss to follow-up

### Clinical pharmacist interventions

Table 4 indicates the interventions of the clinical pharmacists. By the end of the study, 109 interventions had been carried out for 74 patients (52.5%). Patients with serum concentrations of AEDs outside the therapeutic range were more likely to receive medication modifications. Dosage adjustment accounted for the largest proportion (43/109 interventions). In these cases, patients encountering mild or moderate ADRs had their doses decreased, while those with poor seizure control were adjusted to higher doses of AEDs. Patients with uncontrolled seizures or refractory epilepsy were also given additional or alternative drugs with better efficacy (levetiracetam, oxcarbazepine, etc.). Those with major-severe ADRs (i.e., dermatologic or hepatic effects) were either switched to newer generation AEDs with safer profiles (topiramate, levetiracetam, etc.) or discontinued from their current medications. Pharmacists also counseled patients to improve their adherence to the therapy and to instruct initial steps to manage any possible ADRs.

**Table 4:** Interventions by clinical pharmacists (*N* = 109)

	Within therapeutic range (*n* = 47)	Outside therapeutic range (*n* = 62)	*p*-value
Dosage adjustment, *n* (%)	19 (40.4)	24 (38.7)	0.856*
Medication consultation, *n* (%)	7 (14.9)	10 (16.1)	0.860*
Medication switch/discontinuation, *n* (%)	12 (25.5)	15 (24.2)	0.873*
Combination therapy, *n* (%)	9 (19.1)	13 (21.0)	0.815*
Total (*N* = 109), *n* (%)	47 (43.1)	62 (56.9)	0.042*

*Two proportion *Z*-test

### Effectiveness of pharmacist interventions

The effects of the pharmacists’ interventions are reported in Table 5. Before implementing the interventions, 56% of the patients had good seizure control, and the drug serum concentrations of 51.8% of patients were within the therapeutic range. At the study endpoint, 17.7% of the participants were lost to follow-up. The proportion of patients in good control of their seizures had increased significantly to 81%. The TDM for AEDs was carried out again, showing that 66.4% of patients had maintained stable serum concentrations. Given the considerable percentage of loss to follow-up, the best–worst case analyses were conducted. In the worst-case scenario, there were no differences in the drug serum concentration (*p* = 0.633) or in the number of patients with good seizure control (*p* = 0.067). In contrast, in the best-case scenario, the interventions had improved both the treatment effectiveness and the patients’ medication blood levels (*p* < 0.001).

**Table 5:** Analysis of the outcomes at the study endpoint

	Before interventions			After interventions			*p*-value
	Monotherapy (*n* = 99)	Polytherapy (*n* = 42)	Total (*N* = 141)	Monotherapy (*n* = 79)	Polytherapy (*n* = 37)	Total (*N* = 116)	
Treatment effectiveness, *n* (%)							
Good control	61	18	79 (56.0)	66	28	94 (81.0)	< 0.001^*^
Poor control	38	24	62 (44.0)	13	9	22 (19.0)	
Drug serum concentration, *n* (%)							
Within therapeutic range	57	16	73 (51.8)	53	24	77 (66.4)	0.018^*^
Outside therapeutic range	42	26	68 (48.2)	26	13	39 (33.6)	

Worst and best case analysis			Total (*N* = 141)			Total (*N* = 141)	
Worst case							
Treatment effectiveness, *n* (%)							
Good control	–	–	79 (56.0)	–	–	94 (66.7)	0.067*
Poor control	–	–	62 (44.0)	–	–	47 (33.3)	
Drug serum concentration, *n* (%)							
Within therapeutic range	–	–	73 (51.8)	–	–	77 (56.0)	0.633*
Outside therapeutic range	–	–	68 (48.2)	–	–	64 (44.0)	
Best case							
Treatment effectiveness, *n* (%)							
Good control	–	–	79 (56.0)	–	–	119 (84.4)	< 0.001*
Poor control	–	–	62 (44.0)	–	–	22 (15.6)	
Drug serum concentration, *n* (%)							
Within therapeutic range	–	–	73 (51.8)	–	–	102 (73.8)	< 0.001*
Outside therapeutic range	–	–	68 (48.2)	–	–	39 (26.2)	

*Chi-square test, conducted between the total before and after the interventions

## Discussion

Epilepsy management is a complicated process, and the outcomes can be hard to predict. The main target is to achieve a seizure-free status without significant adverse effects [9]. A proper treatment strategy is critical for controlling seizures and for minimizing possible ADRs. This study showed that CBZ (45.4%) and VPA (46.1%) were prescribed more frequently than PHT (18.4%) for epilepsy treatment. In many countries, phenytoin is no longer a first-line therapy due to concerns over its adverse effects [10], which partly explains why it was given less frequently to epileptic patients in this study. Many guidelines now recommend the use of monotherapy with AED for most patients because of similar efficacy and better tolerability compared to polytherapy [11]. As reported in some prior studies, the proportion of monotherapy is pretty high and can be up to 71% [12, 13]. This study’s result also showed a preference for monotherapy in over 70% of the patients. However, there is still a need for evidence-based guidelines for an overall optimal recommended initial monotherapy with AED [14], and further research should be conducted to identify influential factors in the effectiveness of AED monotherapy.

In terms of dosage, there is a noticeable difference between the recommendations of the WHO and the clinical practice in many countries, as reflected in previous publications [12, 15]. The majority of these studies report a lower PDD than the recommended DDD. A reason for this may be that many AEDs can cause dose-related neurotoxic adverse reactions, so to mitigate these effects, monotherapy, with the lowest effective dose, should be applied for all patients whenever possible [16]. Despite this discrepancy, the PDD/DDD ratio (or %DDD) could be a significant indicator of subsequent outcomes. When the seizures of a patient using their first AED at a dose above 50% or 75% of DDD were uncontrolled, the patient had a lower probability of being seizure-free at the last follow-up (*p* < 0.001) [17]. When considering an appropriate dose for defining treatment failure [17, 18], the threshold of either 50% or 75% of DDD could be applied in a definition of refractory epilepsy [17].

In epilepsy treatment, mitigation of ADRs is a critical target for all patients. This study’s results showed that 56.7% of patients experienced ADRs when undergoing AED therapy, a result which is similar to that of a study investigating the ADRs of newer AEDs in Germany, where 56.6% of patients were reported with ADRs [19]. Findings from the literature suggest that newer AEDs may have better tolerability and safety than older generation medications, i.e., carbamazepine, phenytoin, and valproic acid [20]. Despite this, the similarity in prevalence between these two sets of ADR could possibly have resulted from the interventions of the clinical pharmacists in our study, which aimed to minimize the adverse effects of AEDs. Furthermore, the question of whether monotherapy or polytherapy is associated with more ADRs is still controversial [19]. Although in this study, the two types of therapy did not differ significantly in terms of adverse reactions, evidence from previous research indicates a higher risk of side effects in patients on polymedication regimens [16, 21].

Epilepsy management may involve engagement from many sides, including healthcare professionals and family members of the patients. As medication therapy is the most favored approach in controlling seizures [22], the role of the clinical pharmacist is explicitly important. Medical staff usually have to consider the trade-off between epilepsy control and the minimization of possible ADRs. This requires inter-professional involvement if it is to ensure the best treatment for all patients. As a member of the healthcare team, the clinical pharmacist can engage in counseling therapeutic compliance, TDM of AEDs, monitoring drug interactions and ADRs, and adjusting dosage and medications. Based on these functions, this study implemented critical interventions to optimize therapy for patients with epilepsy. In the course of this, dosage adjustment was carried out for 39.4% of patients. This figure is comparable to the results of a study in Thailand (2004), in which 38.9% of patients received pharmacists’ recommendations for adjusting their dosage regimens [23]. About 15% of the patients in our study received additional AEDs due to poor control of seizures and the intolerability of dose-increased medications. Our pharmacists tried to keep this proportion minimal to lower the risks of ADRs to the patients.

The pharmacists’ interventions in this study showed positive changes in epilepsy management and the serum concentration of AEDs. Although 17.7% of the participants were lost to TDM follow-up, after performing a best–worst-case analysis, at the very least, the interventions did not worsen the patients’ drug blood levels or condition. In the more optimistic scenario, most of the patients benefitted from the implementation of the intervention. This finding agrees with previous studies that have stressed the importance of clinical pharmacists being involved in epilepsy management [22, 24].

TDM is a useful tool by which to evaluate the potential causes of toxicity, lack of efficacy, or loss of efficacy [9]. A clinical pharmacist can help optimize this procedure by requesting TDM when necessary, giving instructions on when and how to collect samples for the TDM, and then adjusting dosage based on the TDM results. However, TDM is not a “*golden key*” in managing epilepsy. The therapeutic ranges for AED are derived from the whole population, while individuals might need a lower or higher dose of AED therapy to control their seizures [16]. Therefore, pharmacists should not depend solely on TDM results to adjust the dosage or change the medication, but multiple factors, especially the patient’s response, must also be considered before the final decision is reached [25].

Combining with other findings from some low- and middle-income countries [23, 26–28], we had suggested some strategies to assist clinical pharmacists in these regions to overcome challenges from epilepsy management. First, in addition to regularly counseling patients, pharmacists should also actively engage and implement educational programs to improve patients’ knowledge and perception of epilepsy [26]. Second, pharmacists should focus on routine TDM of AEDs to maximize its benefits and cost-effectiveness [27, 28], which could improve the treatment efficacy and seizure control [23]. Noticeably, as TDM is a multidisciplinary approach [23], this practice can also enhance the inter-professional collaboration among medical staff, thus increasing the overall healthcare quality of the treatment facilities. Finally, in countries where the access to newer AEDs is limited, pharmacists should engage with physicians to optimize the available therapy—either in dosage through TDM or in AED choice through combining/switching medications—to ensure the rational treatment for their patients.

In addition to its helpful findings, this study also has certain limitations. First, the TDM procedure could only be carried out twice for each patient. All other differences in drug concentrations during the study were not recorded, and the final assessment may therefore not be the best reflection of the true effectiveness of the interventions. Second, the outcomes were measured mainly in relation to the number of seizures, while other important factors, such as hospital readmission or quality of life, were not taken into account.

## Conclusion

Management of epilepsy requires many steps, with a focus on the health and safety of the patients. A comprehensive approach should be applied to optimize the treatment for all patients, from monitoring the drug concentration and potential adverse effects to adjusting dosage and switching medications whenever needed. As a part of the healthcare team and with a critical role to play in epilepsy management, pharmacists need to engage in every stage to monitor the patient’s response and to determine the most effective treatment with the fewest ADRs.

## Data Availability

The datasets generated and analyzed during the current study are available from the corresponding author on reasonable request.

## Abbreviations

ADR: Adverse drug reaction
AED: Antiepileptic drug
CBZ: Carbamazepine
DDD: Defined daily dose
NDGD: Nhan Dan Gia Dinh
PDD: Prescribed daily dose
PHT: Phenytoin
TDM: Therapeutic drug monitoring
VPA: Valproic acid
WHO: World Health Organization
